# Prevalence and Outcomes of Candida auris Infections in a Tertiary Hospital in the United Arab Emirates (UAE)

**DOI:** 10.7759/cureus.69988

**Published:** 2024-09-23

**Authors:** Ahmad Subhi, Salma Alshamsi, Aulin Vitus, Akram Harazeen

**Affiliations:** 1 Department of Infectious Diseases, Al-Qassimi Hospital, Sharjah, ARE; 2 Department of Prevention and Control of Infection, Al-Qassimi Hospital, Sharjah, ARE; 3 Department of Internal Medicine, Al-Qassimi Hospital, Sharjah, ARE

**Keywords:** candida auris, candidemia, c. auris, invasive candidiasis, mortality, poor outcome, risk factors

## Abstract

Background

*Candida auris *(*C. auris*) is an emerging serious threat to healthcare settings, with an average mortality of 45% in cases of bloodstream infections. This study aimed to determine the prevalence of *C. auris *in a single center in the UAE during the year 2022 and understand risk factors related to poor outcomes.

Methods

This retrospective cohort chart review at Al-Qassimi Hospital encompassed all confirmed *Candida *infections, including *C. auris*, from January to December 2022. The study involved male and female patients aged 13 years and older, using comprehensive data extracted from the hospital's electronic healthcare records. The analysis included clinical, laboratory, and epidemiological data. Adhering to the 2011 Declaration of Helsinki and Good Pharmacoepidemiology Practices, the study received Institutional Review Board approval, with informed consent waived due to its retrospective design. Data were summarized using appropriate statistical methods, including the unpaired t-test, Mann-Whitney U test, Chi-square test, and Fisher exact test. A significance level of 95% (p<0.05) was maintained throughout the statistical analyses.

Results

Of the 75 confirmed *Candida*infections, 53 (70.7%) were *C. auris*-positive cases. About 23 (43.4%) of the *C. auris *group were above 65 years old. Most cases of *C. auris *group were hospital-acquired (49, 92.5%). The highest number of positive cases were found in urine samples. The demographic and clinical profiles of the *C. auris* and non-*auris* groups candidemia were largely similar, except for differences in antifungal use history and ICU requirements. Notably, the *C. auris *group had a significantly lower history of antifungal use and a lower ICU requirement compared to the non*-auris* group.

The study also highlighted the higher mortality rate associated with candidemia. While mortality was higher in the non-*auris *group, the difference was not statistically significant.

Conclusions

The findings of the study suggest that while *C. auris* poses a serious threat, particularly in hospital settings; the clinical and demographic factors influencing its spread and impact are complex and warrant further investigation. Understanding these factors is crucial for developing effective strategies to prevent and manage *C. auris* infections, particularly in vulnerable patient populations.

## Introduction

*Candida* infection, either bloodstream or deep-seated candidiasis, known as invasive candidiasis, is a significant contributor to morbidity and mortality worldwide, particularly in healthcare settings. One of its causative organisms is the emergent *Candida auris (C. auris)*. Since its first discovery in Japan in 2009, *C. auris* has been linked with causing invasive nosocomial infections associated with considerable mortality rates ranging from 22% to 66% [1,2]. Moreover, one recent systematic review from different countries all over the globe reported an average mortality of 45% (95% CI: 39-51%) for bloodstream *C. auris* infections [3].

*C. auris* is a serious public health concern due to its propensity to spread horizontally and consistently in healthcare settings, including acute and long-term care settings. This is one of its distinctive characteristics. Another noteworthy characteristic is its designation as a developing multidrug-resistant yeast by both US and UK international health bodies [4-6]. As a result, *C. auris* has been identified in hospitals worldwide, with its incidence notably increasing during the coronavirus disease (COVID-19) pandemic [7-9]. This could be attributed to the immunosuppressive effects of COVID-19 and most of its therapeutic armamentarium [10].

The leading cause of the diagnostic difficulty in recognizing *C. auris *strains is the restricted ability of traditional microbiological techniques. Its resistance to certain antifungal medications, such as polyenes, azoles, and echinocandins, further confirms that it poses a serious risk [3,11]. A startling aspect of *C. auris*'s rapid spread is the simultaneous but independent emergence of genetically distinct clades in different regions of the world.

The polygenetic analysis of clinical isolates of *C. auris* collected from various regions in Asia and Africa has revealed four highly clonal phylogenetic and geographically distinct clades that have emerged seemingly independent of one another, specifically, clade I (the South Asian clade), clade II (the East Asian clade), clade III (the South African clade), and clade IV (the South American clade) [9,12,13]. A fifth (Iranian clade) was identified [14-16]. That leads to different antifungal resistance profiles, severely limiting therapy options, therefore, highlighting the critical importance of prevention [17].

The first UAE report of *C. auris* was in a female patient with persistent candidemia who was admitted to Cleveland Clinic Abu Dhabi Hospital in 2018 [18]. The first comprehensive UAE-wide epidemiological analysis of all reported *C. auris* data over four years from 2018 to 2021 was conducted to investigate the trend in the incidence of *C. auris*. The study found a rising incidence of *C. auris* as it reported 908 *C. auris* isolates from 2018-2021 (2018: n=9; 2019: n=93; 2020: n=192; 2021: n=614) [17].

Understanding the epidemiology and risk factors contributing to the burden of* C. auris* is crucial to aid in preventive strategies. Therefore, this current study aims to achieve an in-depth understanding of the epidemiology of *C. auris* infection. The research aimed to determine the prevalence and outcome of *C. auris* infections in patients admitted to our tertiary hospital in 2022.

## Materials and methods

This is a single-center, retrospective cohort chart review study conducted at Al-Qassimi Hospital, UAE. The study adhered to the 2011 Declaration of Helsinki principles and Good Pharmacoepidemiology Practices guidelines. An Institutional Review Board approved the study, and considering the retrospective nature of the study, the need for informed consent was waived. An ethical committee approval was granted.

Al-Qassimi Hospital, the largest government hospital in the UAE, is a critical hub for managing patients at high risk for *C. auris* infections, owing to its extensive and specialized medical services. Departments like intensive care units (ICUs), coronary care units, isolation units, and divisions such as emergency medicine, critical care medicine, cardiothoracic, thoracic, and vascular surgery, general surgery, and orthopedic surgery cater to patients with severe, complex conditions and compromised immunity. This focus on critical care places the hospital at the forefront of managing and preventing *C. auris* infections, emphasizing its role as a leading tertiary care center. The hospital is well-equipped to manage complex cases, making infection prevention a key focus in its operations.

Data collection

*Candida* infection, or candidiasis, can range from superficial, such as oral thrush or vaginal yeast infections, to invasive, where the yeast enters the bloodstream or internal organs, leading to serious systemic infections known as invasive candidiasis. Invasive candidiasis is a more severe form of candidiasis. The infection spreads beyond the superficial layers and involves the bloodstream (candidemia) or other organs such as the brain, bones, liver, or other internal organs. This condition is hazardous in hospitalized patients or those with weakened immune systems, such as individuals with HIV/AIDS, cancer patients undergoing chemotherapy, organ transplant recipients, or those in ICUs.

The definition of *Candida* infection or invasive candidiasis, as used in this study, was based on confirmed cases where patients had positive laboratory results for *Candida* species. These cases were identified through clinical records of male and female patients aged 13 and older admitted to or visiting the outpatient department of Al-Qassimi Hospital between January 1, 2022, and December 31, 2022. The selection of patients focused on those with confirmed *Candida *infections, ensuring accurate identification and inclusion in the study's analysis.

At Al-Qassimi Hospital, the laboratory techniques used to distinguish between different *Candid*a species were comprehensive and multifaceted. Culture-based identification involved growing the organism on selective media to observe colony morphology and growth characteristics. Distinguishing between different types of *Candida* involves a combination of morphological and biochemical methods. Morphological identification includes microscopic examination of stained preparations, allowing for the observation of specific characteristics unique to each *Candida* species. Complementing this, biochemical tests, such as those performed by the VITEK 2 Compact automated system (bioMerieux, Marcy-l'Étoile, France), rapidly and accurately identify *Candida* species through a series of biochemical reactions.

The study's data collection process was robust, with clinical, laboratory, and epidemiological data extracted directly from the hospital's electronic healthcare records. This ensured the accuracy and comprehensiveness of the data. The data extracted from the charts were de-identified following Health Insurance Portability and Accountability Act Privacy Rule guidelines. This involved carefully removing the patient's medical record number, national identification number, and other patient identifiers, ensuring the privacy and confidentiality of the patients [19,20].

The study's data compilation process was comprehensive, with all data on patients' demographic information, baseline features, comorbidities, laboratory results, and clinical outcomes meticulously compiled in an Excel version 365 worksheet (Microsoft Corporation, Redmond, WA, USA). This included detailed information on each patient's age, gender, medical history, and the results of various laboratory tests, providing a comprehensive overview of the patient's health status and outcomes.

The study methodology was comprehensive, comprising several distinctive parts designed to collect diverse data. The audit tool component of the study, for instance, aimed to collect data on various variables of interest, such as demographics, diagnostic tools used, risk factors, and outcomes. That was achieved by administering an appropriate Excel audit tool to the data of study participants as per inclusion criteria. The study team was trained to administer the audit tool and ensure data quality, consistency, and confidentiality. All data from the hospital's electronic record system were cross-checked with the historical records provided by the microbiology laboratory team and the records from the hospital's infection control department. This meticulous approach was taken to obtain the primary dataset using an Excel sheet designed to address the study's objectives, ensuring the thoroughness and reliability of the study's findings.

Exclusion criteria

All records with any of the following were excluded: records with non-*candida* infection, duplicated records, and records with incomplete data (records missing the *Candida* type or all outcome data).

The study adopted an epidemiological definition of hospital-acquired infections as stated by the National Health Safety Network of the USA. This definition considers hospital-acquired infections to be any infection determined after the second calendar admission date. By adhering to this established definition, the study ensures the validity and comparability of its findings, instilling confidence in the audience about the study's conclusions [21,22].

Statistical methods

Descriptive data were summarized as the mean ± standard deviation (SD) for continuous variables normally distributed, median (interquartile range (IQR)) for non-parametric data, or frequency (percentage) for categorical variables. Patient characteristics according to the study cohort were compared using the unpaired t-test or the Mann-Whitney U test in case of continuous variables and the Chi-square test or the Fisher exact test for categorical variables, as appropriate. All statistical tests were carried out using a significance level of 95%. A value of p<0.05 was considered statistically significant. SPSS Statistics version 25.0 (IBM Corp. Released 2017. IBM SPSS Statistics for Windows, Version 25.0. Armonk, NY: IBM Corp.) was used for the statistical analyses.

Outcomes

The primary outcome measure included the prevalence of *C. auris* among all cases of candidiasis. Secondary outcome measures encompassed comparing the different clinical-epidemiologic factors and outcomes between *C. auris* and non-*auris* infection, with emphasis on the candidemia cases.

## Results

At Al-Qassimi Hospital, there were 9,125 cases during the year 2022. The data collection team identified 98 records of cases during this period. After the data check, 23 records were excluded: one record with incomplete data (*Candida* type, all outcome data, hospital stay, all demographic and clinical data apart from gender), four records with no *Candida* infection, and 18 duplicated records, leaving only 75 records included in this study analysis.

Prevalence of *C. auris*

**Figure 1:**
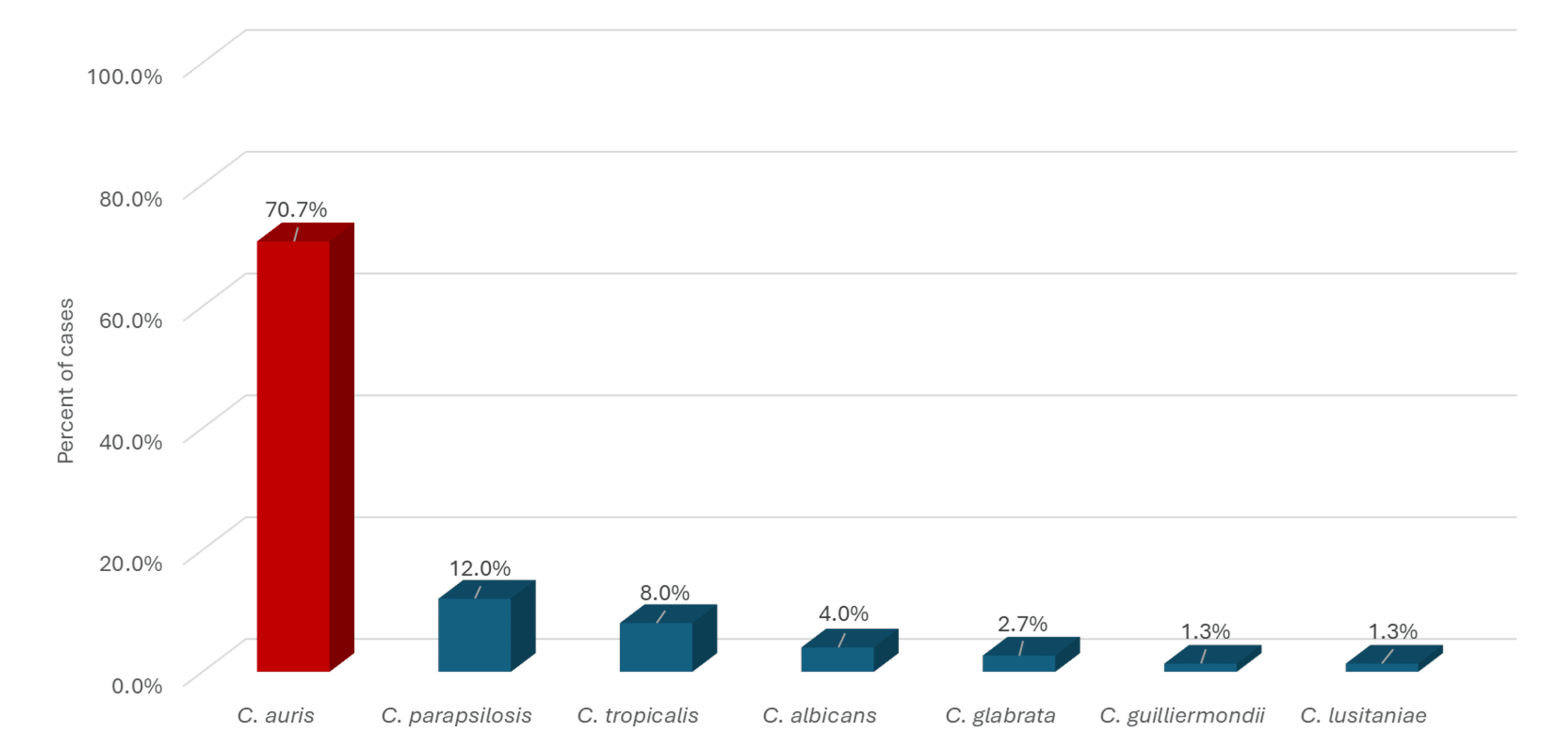
Culture result in the included cases

Out of the 9,125 cases during the year 2022, 75 patients (0.82%) had invasive candidiasis or colonization. *C. auris* was detected in the hospital with a prevalence of 0.58% of all the 9125 cases admitted. As shown in Figure 1, 53 (70.7%) of those 75 patients had *C. auris*, 9 (12.0%) had *C. parapsilosis*, and 6 (8.0%) had *C. tropicalis*.

Description of patients’ characteristics of all *C. auris* and non*-auris* infection (candidemia and colonization)

Both groups were comparable regarding their demographic data (age, gender, and nationality), any comorbid conditions (DM, hypertension, dyslipidemia, cerebrovascular accident, ischemic heart disease, renal impairment/failure, and other conditions), and multi-morbidities with p-values >0.05, as shown in Table 1.

**Table 1:** Patients’ characteristics of those with C. auris versus other Candida isolates Data is presented as n (%) and/or mean ± SD. Statistical tests: unpaired t-test or the Mann–Whitney U test in case of continuous variables and the Chi-square test or the Fisher exact test for categorical variables. A value of p<0.05 was considered statistically significant. AKI: acute kidney injury, CKD: chronic kidney disease, DM: diabetes mellitus, ESRD: end-stage renal disease, HD: hemodialysis, ICU: intensive care unit, IQR: interquartile range, SD: standard deviation, TPN: total parenteral nutrition, *C. auris*: *Candida auris*

Parameter	*C. auris* (N=53)		*Non-auris* (N=22)		p-value
Age >65	23	43.40%	7	31.80%	0.351
Age					
Mean ± SD	57.1 ± 20.4		57.2 ± 17.8		0.976
Median (IQR)	58 (33)		58 (25)		-
Gender					
Male	34	64.20%	15	68.20%	0.738
Female	19	35.80%	7	31.80%	-
Emirati	10	18.90%	4	18.20%	0.611
Hospital-acquired infection	49	92.50%	19	86.40%	0.334
Length of stay before diagnosis median (IQR)	30 (44)		14 (54)		0.247
Already in ICU	39	73.60%	18	81.80%	0.329
Multi-morbidities	25	47.20%	11	50.00%	0.823
DM	24	45.30%	11	50.00%	0.709
Hypertension	21	39.60%	9	40.90%	0.918
Dyslipidemia	7	13.20%	0	0.00%	0.078
Cerebrovascular accident	9	17.00%	3	13.60%	0.508
Ischemic heart disease	11	20.80%	5	22.70%	0.849
ESRD all	3	5.70%	1	4.50%	0.665
ESRD on HD	1	1.90%	1	4.50%	0.503
AKI	4	7.50%	0	0.00%	0.241
CKD	3	5.70%	3	13.60%	0.237
Specimen					
Blood	10	18.90%	22	100.00%	<0.001
Sputum	15	28.30%	0	0.00%	-
Urine	20	37.70%	0	0.00%	-
Wound/others	8	15.10%	0	0.00%	-
Required ICU	43	81.10%	22	100.00%	0.024
Central line	46	86.80%	21	95.50%	0.254
History of antifungal	23	43.40%	15	68.20%	0.051
History of antibiotic	52	98.10%	20	90.90%	0.204
TPN	8	15.10%	2	9.10%	0.388
Invasive procedures during the stay	39	73.60%	13	59.10%	0.215
Length of stay after diagnosis median (IQR)	39 (73)		17.5 (91)		0.762
Mortality	28	52.80%	16	72.70%	0.111

The length of hospital stay before diagnosis of candidal infection was comparable between the two groups (p=0.247). Also, most cases in the *C. auris* group (49, 92.5%) and the non*-auris* group (19, 86.4%) were hospital-acquired infections (p=0.334). In addition, most cases in the *C. auris* group (39, 73.6%) and the non*-auris* group (18, 81.8%) were admitted to a high-risk unit (p=0.329).

As depicted in Table 1, one significant difference (p<0.001) was noticed in all the isolates of the non-*auris* group detected in blood, where only 18.9% of the *C. auris* group was isolated from blood specimens, 37.7% from urine, 28.3% from sputum, and 15.1% from wounds, pleural fluid, and abscesses.

All cases of the non-*auris* group were candidemia cases, in contrast to the *C. auris* group, which has only 18.9% candidemia; therefore, for a better understanding of the comparability of the outcomes of *C. auris* and non-*auris* cases, the analysis in the following section will be restricted only to candidemia cases.

Comparison between *C. auris* and non-*auris* candidemia cases

Out of all cases, only 10 (18.9%) cases of the 53 cases with *C. auris*, and 22 (100.0%) of the non-*auris* cases had candidemia. As depicted in Table 2, among all the different demographic and clinic-pathological factors studied, only the history of antifungal use and the ICU requirement were statistically different between *C. auris* candidemia and non-*auris* candidemia groups. There is a statistically significant difference between *C. auris* (10.0%) and non-*auris* (68.2%) groups with regard to the history of antifungal use, with a p-value of 0.003. A significant difference is observed in the ICU requirement between *C. auris* candidemia (60.0%) and non-*auris* candidemia (100.0%) groups, with a p-value of 0.006. Other variables did not show statistically significant differences between the groups. No significant difference was seen with regard to the mortality between groups.

**Table 2:** Patients’ characteristics of those with C. auris versus non-auris candidemia Data is presented as n(%) and/or mean ± SD or median (IQR). Statistical tests: unpaired t-test or the Mann–Whitney U test in case of continuous variables and the Chi-square test or the Fisher exact test for categorical variables. A value of p<0.05 was considered statistically significant. AKI: acute kidney injury, CKD: chronic kidney disease, DM: diabetes mellitus, ESRD: end-stage renal disease, HD: hemodialysis, ICU: intensive care unit, IQR: interquartile range, SD: standard deviation, TPN: total parenteral nutrition

Parameter	*C. auris* (N=10)		*Non-auris* (N=22)		p-value
Age >65	4	40.00%	7	31.80%	0.474
Age (mean + SD)	59.6	14.3	57.2	17.8	0.715
Length of stay before diagnosis as candidemia (median + IQR)	25	32	14	54	0.919
Length of stay after diagnosis as candidemia (median + IQR)	6	12	17.5	91	0.07
Gender					
Female	2	20.00%	7	31.80%	0.405
Male	8	80.00%	15	68.20%	-
Emirati	2	20.00%	4	18.20%	0.627
Multi-morbidity	7	70.00%	11	50.00%	0.253
DM	7	70.00%	11	50.00%	0.253
Hypertension	5	50.00%	9	40.90%	0.459
Cerebrovascular accident	2	20.00%	0	0.00%	0.091
Ischemic heart disease	2	20.00%	3	13.60%	0.506
ESRD all	2	20.00%	1	4.50%	0.224
ESRD on HD	0	0.00%	1	4.50%	0.688
AKI	1	10.00%	0	0.00%	0.313
CKD	0	0.00%	3	13.60%	0.31
Dyslipidemia	0	0.00%	1	4.50%	0.688
History of antifungal	1	10.00%	15	68.20%	0.003
History of antibiotic	9	90.00%	20	90.90%	0.69
Already in ICU	7	70.00%	18	81.80%	0.376
Invasive procedures during the stay	8	80.00%	13	59.10%	0.229
Central line	8	80.00%	21	95.50%	0.224
TPN	1	10.00%	2	9.10%	0.69
Hospital-acquired infection	8	80.00%	21	95.50%	0.224
Required ICU	6	60.00%	22	100.00%	0.006
Mortality	6	60.00%	16	72.70%	0.373

## Discussion

The distribution of *Candida *species has changed significantly over the recent years, with a noticeable decrease in the prevalence of *C. albicans *and an increase in non-*albicans* subspecies, including *C. auris*. In this study, among the 75 patients admitted during the study period with *Candida* infections (including candidemia and colonization), the proportion of C.* auris *was notably high at 53 (70.7%). This figure is considerably higher than previously reported data from the UAE, where the proportion of *C. auris* among all *Candida* infections was 0.39% in 2018, 2.92% in 2019, 5.01% in 2020, and 4.73% in 2021 [17]. The rising trend in *C. auris* infections raises critical questions regarding the factors driving this increase, underscoring the urgent need for further investigation.

Our findings are in line with reports from other countries, confirming the widespread prevalence of *C. auris*. A systematic review covering January 2019 to January 2021 reported *C. auris* cases in several regions: 71 cases in Kuwait, 29 in Oman, 35 in Saudi Arabia, 47 in Spain, and 12 each in Mexico and Kenya [13]. These results underscore the global nature of *C. auris* spread, highlighting the need for a coordinated international response.

In our study, males constituted 64.2% of the *C. auris* group and 68.2% of the non-*auris* group. These figures are higher than those reported in the UAE over the four years, where 52.5% of the *C. auris* group, and 25.4% of the non-*auris* group were males [17]. However, our findings are consistent with studies from Saudi Arabia (62.3% male) [21] and a worldwide retrospective analysis from 2009 to 2020 (61.4% male) [23].

The mean age in our study was 57.1 ± 20.4 years for *C. auris* cases and 57.2 ± 17.8 years for non-*auris* cases, with no statistically significant difference (p=0.976). That was in contrast to the Saudi research that reported a median age of 64 (IQR 15-98) for the *C. auris* group [21]. In our cohort, 43.4% of the *C. auris* group were over 65 years old, higher than the 37% reported in an Omani study [24]. Other studies also showed that nearly half of the cases were in patients around 70 years old [25,26].

Regarding nationality, only 18.9% of the *C. auris* group, and 18.2% of the non-*auris *group were UAE nationals. That is lower than the 29% of nationals reported in the earlier UAE study [17].

The isolation of *C. auris *in this study was most frequent from urine samples (37.7%), followed by sputum (28.3%), blood (18.9%), and wounds/other sites (15.1%). In contrast, all non-*auris* isolates came from blood samples. This distribution differs slightly from the UAE four-year data, which reported *C. auris* isolation from urine (30.8%), respiratory tract (15.6%), blood (27.3%), and skin/soft tissue (24.3%) [17]. Similarly, in a study from Saudi Arabia, urine samples yielded the highest number of cases (30.2%), followed by samples from the thigh, buttock, and hip (18.8%) and axilla (11.3%), with only one blood sample [21]. Another study reported blood (38.9%) and urine (36.3%) as the most common sample sources [24]. These findings suggest that physicians should consider sites other than blood when isolating *C. auris*.

Our analysis indicated that in most cases, 92.5% of the *C. auris* group, and 86.4% of the non-*auris* group were hospital-acquired. In the earlier UAE study, 89% of *C. auris* cases were also hospital-acquired [17]. Additionally, 73.6% of *C. auris* patients and 81.8% of non-*auris* patients had been admitted to high-risk units. That is higher than the 35.8% reported in the Saudi study [21] but comparable to a multinational study, which found that 72% of the *C. auris* cases were associated with high-risk units [26]. In the UAE study, 45.6% of *C. auris* cases were related to high-risk units [17].

None of the risk factors we examined predicted *C. auris* infection in particular. However, ICU admission was significantly higher in the non-*auris* group (100%) compared to the *C. auris* group (81.1%) (p=0.024). When analyzing candidemia cases alone, we found that the history of antifungal use and ICU requirement statistically differed between groups. Antifungal use was much more common in non-*auris* candidemia cases (68.2%) compared to *C. auris *candidemia (10.0%) (p=0.003). The ICU requirement was also higher in non-*auris* candidemia cases (100%) than in *C. auris* candidemia cases (60.0%) (p=0.006).

A previous study reported that recent antibiotic treatment (96%), antifungal therapy (28%), and total parenteral nutrition (22%) were common therapeutic risk factors for acquiring* C. auris *[26]. Mortality in candidemia cases is highly dependent on the specific patient population. Many patients have underlying medical conditions, making it challenging to attribute mortality solely to *C. auris* infection versus other causes. Mortality is generally reported as 30-day all-cause mortality. Our study reported mortality rates of 60% in the *C. auris* group and 72.7% in the non-*auris* candidemia group, both higher than those reported in the earlier UAE study (27.5% and 26.4%) [17].

Our facility lacks accurate antifungal susceptibility testing for *C. auris*, so the standard recommendation for managing *C. auris* candidemia has been a combination of two antifungal agents. The cornerstone of this treatment is liposomal amphotericin B, paired with an echinocandin, selected based on liver function, availability, and the risk of drug-drug interactions.

Limitations

This study has several limitations. First, it is retrospective rather than prospective. Second, the small sample size may limit the generalizability of the findings, though it represents the total number of cases at our center. Third, the study did not report the drug resistance profile of the isolates. These limitations highlight the need for a comprehensive, multicenter prospective study to address these gaps and further understand the spread and impact of *C. auris.*

## Conclusions

The study sheds light on the prevalence and characteristics of *C. auris* infections compared to non-*auris* *Candida* species, highlighting several significant findings. The prevalence of *C. auris* was relatively low, with a hospital prevalence of 0.58%, but it constituted the majority (70.7%) of the 75 candidiasis cases. The demographic and clinical profiles of the *C. auri*s and non-*auris *groups candidemia were largely similar, except for differences in antifungal use history and ICU requirements. Notably, the *C. auris* group had a significantly lower history of antifungal use and a lower ICU requirement compared to the non-*auris* group.

The study also highlighted the higher mortality rate associated with candidemia. While mortality was higher in the non-*auris* group, the difference was not statistically significant. This indicates that candidemia in general, regardless of the specific *Candida* species, poses a significant risk of mortality, particularly in critically ill patients. The study’s findings underscore the need for heightened vigilance in managing candidemia, particularly in high-risk populations, and suggest that further research is needed to better understand the factors contributing to the spread and severity of *C. auris* infections.

The findings suggest that while *C. auris* poses a serious threat, particularly in hospital settings, the clinical and demographic factors influencing its spread and impact are complex and warrant further investigation. Understanding these factors is crucial for developing effective strategies to prevent and manage *C. auris* infections, particularly in vulnerable patient populations.
